# Specificity and diversity of *Klebsiella pneumoniae* phage-encoded capsule depolymerases

**DOI:** 10.1042/EBC20240015

**Published:** 2024-12-17

**Authors:** Max J. Cheetham, Yunlong Huo, Maria Stroyakovski, Li Cheng, Daniel Wan, Anne Dell, Joanne M. Santini

**Affiliations:** 1 Department of Structural and Molecular Biology, Division of Biosciences, University College London, London, WC1E 6AA, U.K.; 2 Department of Life Sciences, Imperial College London, London, SW7 2AZ, U.K.

**Keywords:** capsular polysaccharide depolymerases, capsule, depolymerase, Klebsiella pneumoniae, phage

## Abstract

*Klebsiella pneumoniae* is an opportunistic pathogen with significant clinical relevance. *K. pneumoniae*-targeting bacteriophages encode specific polysaccharide depolymerases with the ability to selectively degrade the highly varied protective capsules, allowing for access to the bacterial cell wall. Bacteriophage depolymerases have been proposed as novel antimicrobials to combat the rise of multidrug-resistant *K. pneumoniae* strains. These enzymes display extraordinary diversity, and are key determinants of phage host range, however with limited data available our current knowledge of their mechanisms and ability to predict their efficacy is limited. Insight into the resolved structures of *Klebsiella*-specific capsule depolymerases reveals varied catalytic mechanisms, with the intra-chain cleavage mechanism providing opportunities for recombinant protein engineering. A detailed comparison of the 58 characterised depolymerases hints at structural and mechanistic patterns, such as the conservation of key domains for substrate recognition and phage tethering, as well as diversity within groups of depolymerases that target the same substrate. Another way to understand depolymerase specificity is by analyzing the targeted capsule structures, as these may share similarities recognizable by bacteriophage depolymerases, leading to broader substrate specificities. Although we have only begun to explore the complexity of *Klebsiella* capsule depolymerases, further research is essential to thoroughly characterise these enzymes. This will be crucial for understanding their mechanisms, predicting their efficacy, and engineering optimized enzymes for therapeutic applications.

## Introduction

*Klebsiella pneumoniae* is a gram-negative, often encapsulated opportunistic bacterial pathogen member of the *Enterobacteriaceae*, implicated in diseases such as pneumonia, pyogenic liver abscesses, and neonatal sepsis [1–3]. In the past decade, a rise in multidrug-resistant (MDR) and hypervirulent (hv) *K. pneumoniae* strains has led to increased infection burden, and its spread among the healthy population [4]. In low- and middle-income countries, *K. pneumoniae* infections are becoming increasingly difficult to treat due to the rapid acquisition of extended-spectrum beta-lactamases, posing a severe threat to public health [5]. The recent emergence and spread of carbapenem-resistant strains have led to elevated concern [2], with the World Health Organisation listing carbapenem-resistant *Enterobacteriaceae* as a priority 1 critical pathogen for the development of novel antimicrobials [6].

The rise of MDR bacteria has sparked renewed interest in bacteriophage (phage) therapy research. While the high host-specificity of these viruses is favourable for the benefit of the patient’s microbiome, challenges arise when producing broad-spectrum therapeutics [7]. Phages initiate infection by recognising and binding to specific cell-surface structures, including porins, pili, lipopolysaccharides (LPS, containing the O-Antigen), and capsular polysaccharides (CPS, or the K-Antigen) [8]. The CPS surrounding the bacterial surface is important for *K. pneumoniae* infection protecting the cell from environmental factors, including antimicrobials [9], and the host immune system [10]. The capsules of *K. pneumoniae* are, therefore, major virulence factors [10]. For many *Klebsiella* phages, recognition, binding, and degradation of the CPS is necessary for successful infection, making the CPS a common first (reversible) receptor and a key determinant of phage host range [11–14].

To date, at least 134 capsule synthesis loci (K-loci) have been identified although only 79 structures have been reported [15]. Capsule serotypes vary in sugar moieties and glycosidic linkages within the repeat units. In response to the physical barrier presented by the capsule, many phages possess receptor binding proteins (RBPs), such as tail spikes or tail fibres, with depolymerase activity. These degrade the CPS and permit cell wall binding and injection of the nucleic acid [15]. These depolymerases demonstrate exceptional capsular specificity and most depolymerases only catalyse a single CPS serotype [16,17]. Capsule removal by phage depolymerases subjects *K. pneumoniae* to complement-mediated killing *in vivo* [18,19] and can improve antibiotic action, such as in the cases of ciprofloxacin, gentamicin, and polymyxin [20–24]. Capsule removal, therefore, presents a promising novel (or combination) treatment for MDR *K. pneumoniae*-induced infections.

Phage encoded depolymerases targeting bacterial polysaccharides (CPS, EPS, and LPS) have been reviewed previously [25,26] and have been shown to share common structural features. A large number of *Klebsiella* phages have been predicted to encode depolymerases (based on the indicative halo plaque morphology and sequence identity), but to date only 58 have been experimentally validated (defined as heterologously expressed with confirmed activity, as opposed to other methods of extraction [27–29]). To date, eight have been structurally characterised, resulting in limited knowledge of the mechanisms underlying CPS recognition and binding. The use of phages or depolymerases to combat *Klebsiella* infections is restricted by the aforementioned diversity and specificity, generating interest in host range engineering. Previous studies have successfully manipulated phage host range by swapping depolymerase domains [12] or by engineering the phages with additional depolymerases [30,31]. The potential for further optimisation of depolymerase activity has been suggested through the engineering of smaller, chimeric multi-domain enzymes [32,33], requiring deeper understanding of enzyme-substrate interactions.

Herein, we review all 58 experimentally validated *K. pneumoniae* phage CPS depolymerases (hereafter named depolymerases) and, (1) compare depolymerase biochemistry and their mechanisms of action, (2) use identified CPS cleavage sites and structures to review the importance of capsule chemistry in determining depolymerase-capsule interactions, and (3) group the enzymes based on their structural diversity and capsule specificity. We focus on the interplay between structure and depolymerase specificity to shed light on possible avenues for engineering multi-serotype specific depolymerases, as well as improving *in silico* activity prediction, to further advance potential phage therapies against *K. pneumoniae*. We also highlight the current challenges and limitations in this field.

## Structural and mechanistic properties of *Klebsiella* CPS depolymerases

### *Klebsiella* depolymerases adhere to typical homotrimeric architecture, but other oligomeric states can also be active

**Figure 1:**
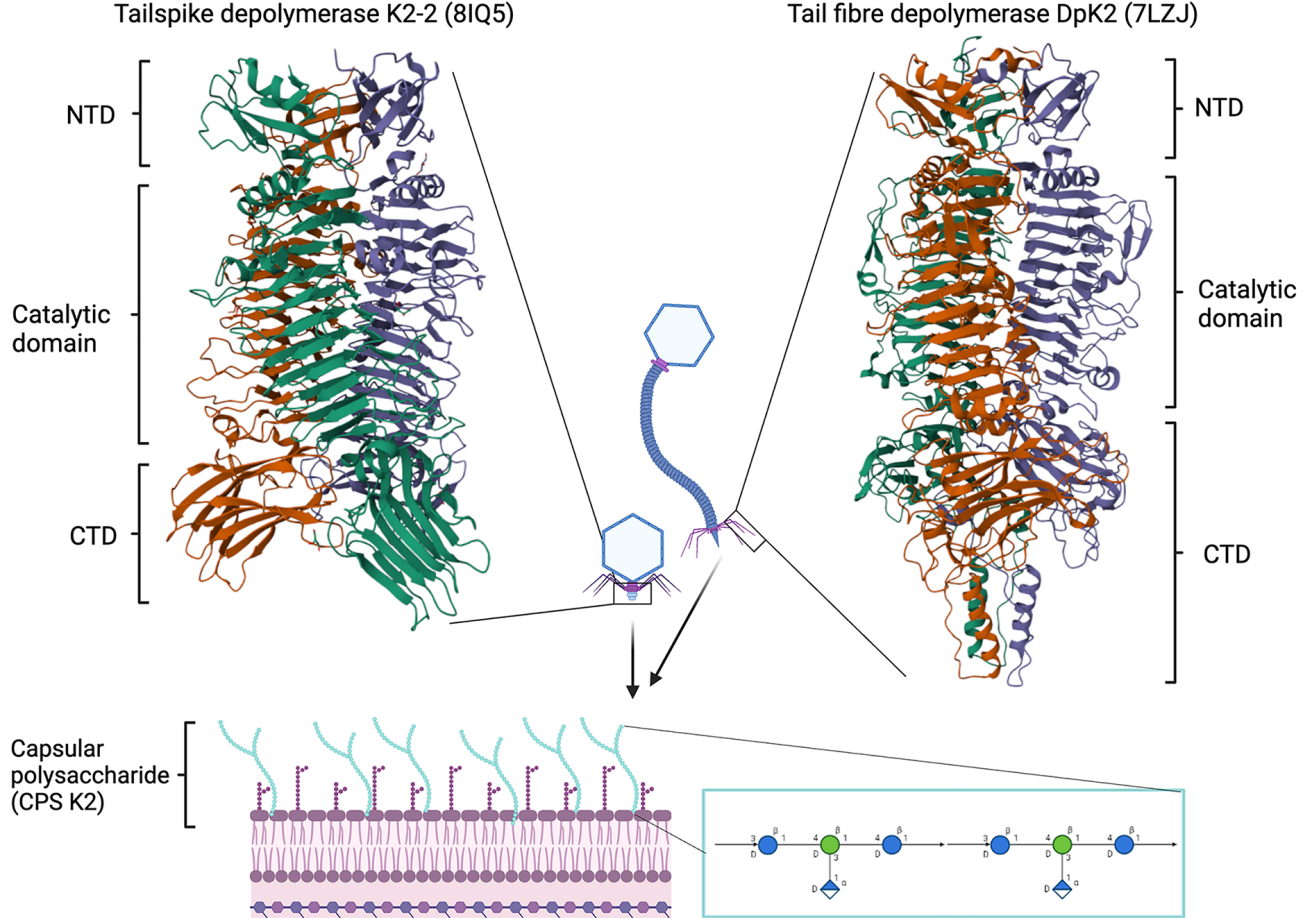
Representation of CPS removal by phage-derived depolymerases *K. pneumoniae* is surrounded by a capsule polysaccharide (CPS) layer (structural representation shown in blue) serving to protect from antibiotics and the immune system. The CPS is composed of various sugars, with the K2 variant illustrated in the figure. Phages can encode depolymerases to target and degrade the CPS for removal. These depolymerases are usually found on the phage tailspike or tail fiber proteins. Two structurally characterised depolymerases, K2-2 (8IQ5) and DpK2 (7LZJ), are depicted in the figure on the tailspike or tail fibre. PDB entries correct at time of writing.

**Figure 2:**
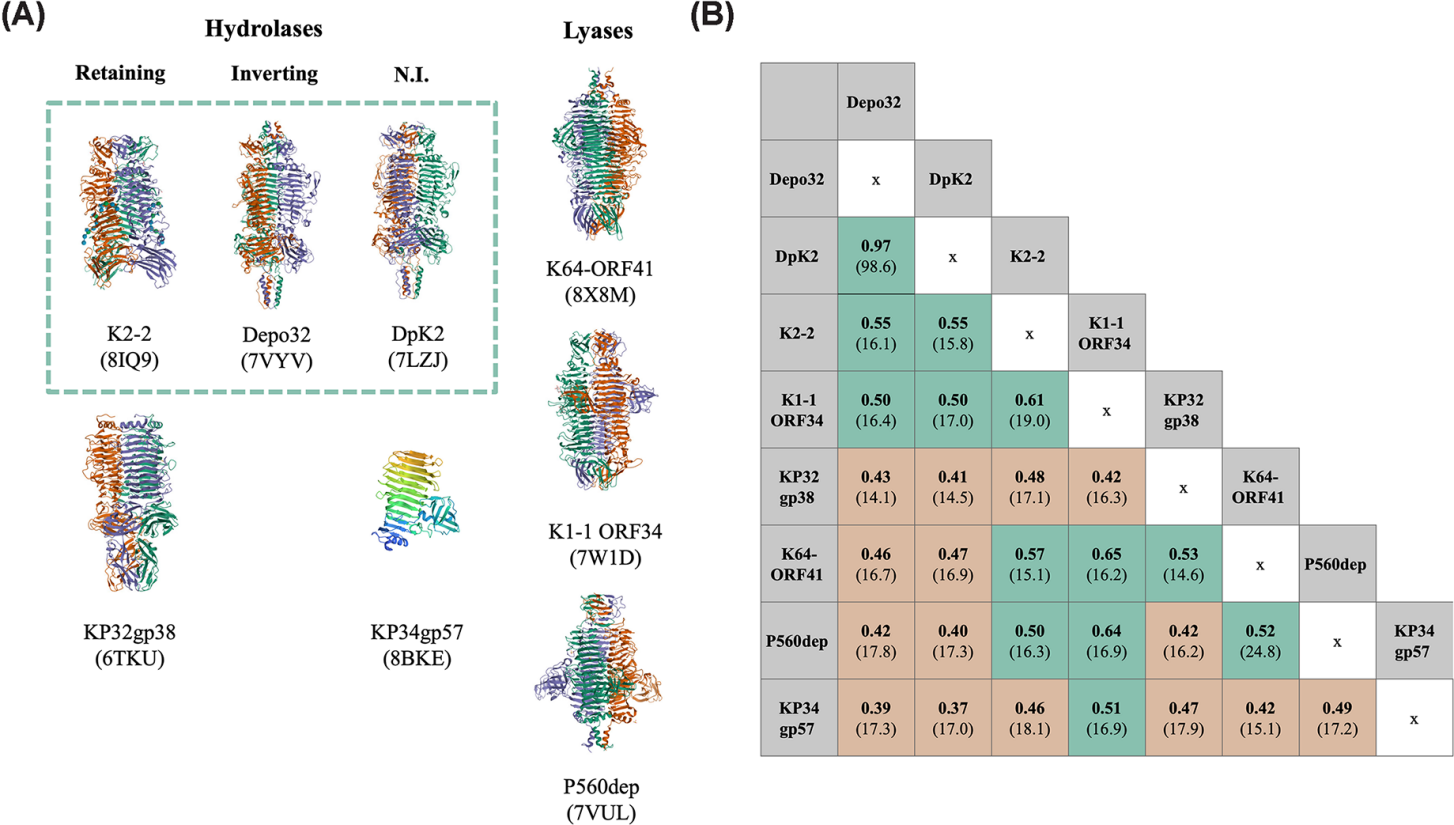
Structure illustrations and TM-scores of eight structurally characterised depolymerases (**A**) The tertiary structures for K2 depolymerases Depo32, DpK2 and K2-2, K1 depolymerase NTUH-K2044-K1-1 ORF34 (K1-1 ORF34 for short), K21 depolymerase KP32gp38, K47 depolymerase P560dep, K64 depolymerase K64-ORF41 and K63 depolymerase KP34gp57 (which was deposited as a truncated monomer). All structures sourced from PDB, with IDs shown in parentheses below. Proteins are oriented NTD to CTD from top to bottom and grouped by depolymerase mechanism. Green box highlights K2 depolymerases. (**B**) Table displaying the TM-score (template modelling score, which ranges from 0-1. Score <0.3 indicates likely unrelated structures and score >0.5 indicates a high probability of proteins with the same fold [65], calculated using TM-align [66]) between these depolymerases. The amino acid sequence identities are listed in parentheses under the TM-score (calculated using EMBOSS Stretcher [67]). Each alignment score is calculated using the depolymerase in the top (cascading) row as the template and cell colours are based on TM-score significance.

Phage depolymerases typically exist as homotrimers, with monomers composed of three functional domains (Figure 1). The N-terminal domain (NTD) tethers the depolymerase to the phage baseplate or tethering protein (which, in some cases, can be another depolymerase [15,34]). The central domain is the site of polysaccharide depolymerisation and adopts a right-handed beta-helix conformation. The catalytic pocket of phage depolymerases can generally be placed between two beta-helices of two neighbouring monomers (inter-subunit location) or between two protruding loops of the same monomer within the homotrimer (intra-subunit) [32]. The protruding shape of the parallel beta-helix topology has been proposed to create a long lateral surface for specific polysaccharide sequence detection, with several consecutive sugars making contact with the protein [35,36]. The C-terminal domain (CTD) is essential for depolymerase trimerisation and is thought to aid in receptor recognition and binding [34]. Several depolymerases contain an auto-cleavable chaperone domain to ensure correct trimerisation [15,36]. At the time of writing, there are eight structurally characterised, experimentally validated *Klebsiella*-specific phage-encoded depolymerases (Table 1), as determined by cryo-electron microscopy (EM) and X-ray crystallography. The structures of these enzymes adhere to the typical homotrimeric architecture (Figure 2A) (for KP34gp57 only the monomeric structure was available); similarities between them are also shown (Figure 2B). Investigations into the catalytic pocket of *Klebsiella* depolymerases demonstrated that catalysis occurs at the inter- and intra-subunit locations [18,32,36], with three of the depolymerases using intra-chain clefts and four using inter-chain clefts (one remains unknown at the time of writing) (Table 1). One study showed the crystal structures of depolymerase K2-2 in complex with hydrolysed K2 CPS product, revealing that the inter-subunit carbohydrate binding grooves accommodated three tetrasaccharide repeating units, similar to those reported for previously characterised phage depolymerases Sf6 and phiAB6 that target *Shigella flexneri* and *Acinetobacter baumannii*, respectively [37–39].

**Table 1:** Structurally characterised *K. pneumoniae* phage depolymerases

Depolymerase	Depolymerase accession number	PDB entry ID	CPS serotype	Activity	Catalytic cleft location	Catalytic amino acids	Reference
**NTUH-K2044-K1-1 ORF34**	YP_009098385.1	7W1D	K1	Lyase - Beta Elimination	Intrachain	Tyr311, His373, Arg397	[18]
**DpK2**	QUU29414.1	7LZJ	K2	Hydrolase - N.I	Interchain	N.I^*^	[8]
**Depo32**	YP_009820105.1	7VYV	K2	Hydrolase - Inverting	Interchain	Glu545/Glu423, Asp546/Glu423	[19]
**K2-2**	QJI52632	8IQ5	K2, K13	Hydrolase - Retaining	Interchain	Glu267/Glu363	[39]
**KP32gp38**	YP_003347556.1	6TKU	K21	Hydrolase - Retaining	Interchain	Asp241/Glu170	[71]
**P560dep**	QOV05501.1	7VUL	K47	Lyase - Beta Elimination	N.A	N.A	to be published
**KP34gp57**	YP_003347651.1	8BKE	K63	Hydrolase - N.I	Intrachain	Glu266/Glu300	[32]
**K64-ORF41**	AUV61507.1	8X8M	K64	Lyase - Beta Elimination	Intrachain	Tyr528, His574, Arg628	[33]

All seven structurally characterised* K. pneumoniae* phage depolymerases are shown, ordered by CPS target. Summary includes CPS serotype, catalytic activity, catalytic cleft location and confirmed catalytic amino acids. Accession numbers provided correct for NCBI GenBank at time of writing. Entry IDs for Protein Data Bank (PDB) correct at time of writing. N.I = Not Identified. N.A = Not Available.

* Catalytic residues for DpK2 were predicted (Glu543, Asp545, Glu546 and Asp399, Glu423 and Asp619, Glu620) but not confirmed via mutational analysis.

Depolymerase trimerisation was until recently thought to be essential for enzymatic function. It was shown, however, that K63-targeting depolymerase KP34gp57 functions efficiently as a monomer following proteolytic cleavage of the CTD, preventing trimerisation [32]. This demonstrated the catalytic activity of the intra-chain cleft, formed between the monomer and a protruding beta-barrel domain, which operates as the main site of catalysis in some homotrimeric depolymerases [18,32,39]. Similar observations were made with monomeric K1 depolymerase NTUH-K2044-K1-1 ORF34, although enzyme activity dropped to 20% of wild-type [18]. Functional intra-subunit catalysis without trimerisation has also been demonstrated for a tetrameric form of the depolymerase K2-2 [39]. Studies have shown that the inter-chain cleft may sometimes act as a saccharide binding pocket, rather than the catalytic cleft. In these cases, the inter-chain saccharide-binding pocket is shown to be essential for depolymerase activity, and has been hypothesised to stabilise reaction intermediates, possibly explaining the reduced activity of monomeric NTUH-K2044-K1-1 ORF34 [18]. This is supported by insight into the substrate binding domain of K64-ORF41, shown to be located on the surface groove on the outer periphery of the catalytic residues [33]. Key residue mutations were shown to decrease enzymatic activity by altering the electrostatic potential, likely affecting CPS binding. Detailed understanding of catalytic activity is key for engineering optimised depolymerases, such as mini-enzymes, for biotechnological or medical applications.

### *Klebsiella* CPS depolymerases have diverse modes of action

**Figure 3:**
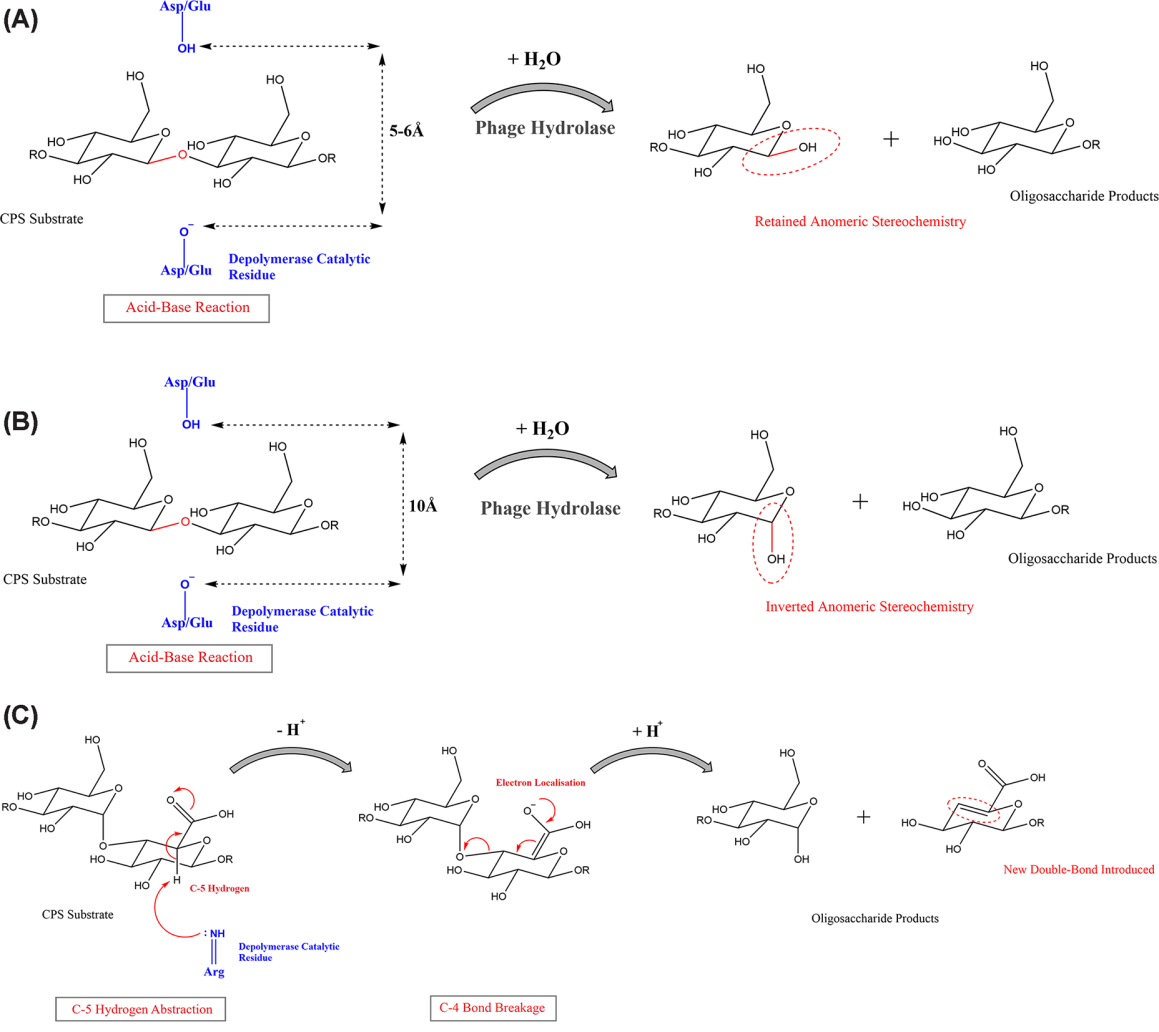
Polysaccharide depolymerase mechanisms of action Depolymerase mechanisms employed by hydrolases are shown in A (retaining) and B (inverting). The mechanism employed by lyases is shown in C. (**A**) Capsule polysaccharide cleavage by the retaining mechanism needs a gap of 5-6Å between the carboxyl sidechains of Asp/Glu catalytic residues, within the depolymerase active site (shown by dashed arrows). The acid-base reaction, requiring the presence of water, results in net retention of anomeric stereochemistry (circled in red) at the cleavage site. (**B**) The inverting mechanism requires a gap of 10Å between the carboxyl sidechains of Asp/Glu catalytic residues (shown by dashed arrows). The process results in inversion of anomeric stereochemistry (circled in red). (**C**) The lytic mechanism of polysaccharide cleavage does not require a water molecule, unlike the hydrolytic mechanisms. The uronic acid allows electron localisation on the carboxyl group following C5 hydrogen abstraction by the catalytic base within the depolymerase active site (shown here as an arginine residue in blue). The C4 linkage is broken, resulting in the introduction of a double bond (circled in red) to the oligosaccharide product.

According to their mode of action, phage depolymerases fall into two main classes: hydrolases (EC 3) and lyases (EC 4), which can be further divided into subclasses [40] (Figure 3). Most often, CPS depolymerases are hydrolases, while LPS depolymerases are lyases [25]. At the time of writing, there are eight structurally characterised, experimentally validated *Klebsiella* phage-encoded CPS depolymerases (Table 1), consisting of five hydrolases, and three lyases. Hydrolases use a water molecule to cleave glycosidic bonds via general acid catalysis, requiring a proton donor and a nucleophile/base. This can result in either inversion or net retention of anomeric stereochemistry [41]. The position of the proton donor remains identical for both retaining and inverting mechanisms however the nucleophile is either close to the sugar anomeric carbon (retaining) or further away (inverting), allowing for mechanistic prediction by measuring the distance between catalytic residues (Figure 3A,B). For example, measuring the distance between two carboxyl sidechains of catalytic AAs in the structurally characterised K2-depolymerase Depo32, demonstrated that it was operating an inverting hydrolytic mechanism at the inter-chain cleft (10Å; the retaining mechanism needs 5-6Å) [19]. Most hydrolases belong to the group of O-glycosyl hydrolases (EC 3.2.1), specifically cleaving the O-glycosidic bonds of the polysaccharide [36]. As an example, among 11 CPS cleavage sites-determined for *Klebsiella* phage depolymerases, nine hydrolyse by acting on β(1→4) or β(1→3) glycosidic bonds, which is the typical cleavage site of O-glycosyl depolymerases [42] (Table 3). Conversely, lyases cleave O-glycosidic bonds via beta-elimination, removing the hydrogen atom from the fifth carbon to break the glycosidic bond on the fourth carbon (Figure 3C). This mechanism requires electron stabilisation on the carboxyl group, thus requiring the sugar to be a uronic acid. Two chemically distinct ends are yielded: an unsaturated sugar on the new non-reducing end with a double bond between C4 and C5, and a saturated sugar on the new-reducing end [35,43,44]. Polysaccharide lyases (EC 4.2.2) cleave only on the non-reducing side of uronic acids [35].

## Specificity of *Klebsiella* phages and their depolymerases

### Depolymerases typically constitute the primary determinant of *Klebsiella-*phage host range

Due to intimate co-evolution, tightly coupled interactions with some degree of specificity between phages and their bacterial hosts are readily observed in nature. In the case of *K. pneumoniae*, polysaccharide diversity is one of the main factors restricting phage host range, and therefore polysaccharide depolymerases are crucial determinants of phage host tropism [45]. Understanding depolymerase specificity is therefore key to predicting phage host range, as illustrated by computational methods determining phage-host specificity at the strain level [46]. Phages can also encode more than one depolymerase, allowing for expanded host range. This is best illustrated by jumbo phage K64-1, which encodes 11 RBPs, nine of which display CPS depolymerase activity, the most of all known *K. pneumoniae*-targeting phages [17]. Similarly, phage vB_KpnM-20 encodes four RBPs, with three having enzymatic activity against known CPS targets [47]. Phages IME205, KP32, K5-4 and KN3-1 encode two depolymerases, while phage K5-2 was predicted to encode two depolymerases, with only one experimentally validated [16]. The remaining phages encode a single depolymerase (Table 2). In all cases, the encoded depolymerases have non-overlapping host ranges, suggesting that the acquisition of multiple capsule depolymerases may confer an evolutionary advantage, especially in response to environments that contain diverse bacterial capsule types. The validated depolymerases target a total of 31 CPS serotypes (Table 2) and range from 555 to 1294 AA in length. Due to clinical relevance, studies have identified a number of different depolymerases against serotypes K1, K2, K20, K54 and K57, associated with hypervirulence, which means they are likely over-represented in the dataset [34].

**Table 2:** Experimentally validated *K. pneumoniae* phage CPS depolymerases

Phage	Phage accession number	Phage family	Genome size (bp)	GC%	Depolymerase	Depolymerase accession number	CPS serotype	Protein length (aa)	Reference
**Kpv71**	KU666550	*Autographiviridae*	43,267	54.0	**Kpv71_52**	YP_009302756.1	K1	651	[55]
**NTUH-K2044-K1-1**	NC_025418.1	*Autographiviridae*	43,871	54.2	**K1-ORF34**	YP_009098385.1	K1	651	[18]
**GBH001**	OU509534.1	*Autographiviridae*	44,964	54.2	**GBH001_056**	N.A	K1	651	[54]
**ZK1**	OK625527.1	*Autographiviridae*	45,839	51.5	**Depo16**	UFK26377.1	K1	794	[56]
**K64-1**	NC_027399.1	*Alcyoneusvirus*	346,602	31.7	**S2-4**	BAW85698.1	K1	888	[17]
**GBH0038**	OU509533	*Autographiviridae*	43,726	54.3	**GBH038_054**	N.A	K2	577	[54]
**1611E-K2-1**	MG197810.1	*Jedunavirus*	47,797	48.9	**K2-ORF16**	ATS92567.1	K2	668	[72]
**NPat**	OM938991.1	*Drexlerviridae*	48,715	50.6	**NPatgp22**	UPW42650.1	K2	763	[73]
**BMac**	OM938992.1	*Drexlerviridae*	48,957	50.2	**BMacgp22**	UPW42726.1	K2	907	[73]
**RAD2**	MW655991.1	*Drexlerviridae*	49,276	50.4	**DpK2**	QUU29414.1	K2	907	[8]
**GH-K3**	NC_048162.1	*Drexlerviridae*	49,427	50.2	**Depo32**	YP_009820105.1	K2	907	[19]
**KpV74**	KY385423.1	*Autographiviridae*	44,094	54.1	**Kpv74_56**	APZ82768.1	K2, K13	577	[14]
**VLC6**	MT197176.1	*Autographiviridae*	44,294	54.2	**K2-2**	QJI52632	K2, K13	577	[39,59]
**B1**	MW672037.1	*Drexlerviridae*	50,040	50.4	**B1dep**	QTP95996.1	K2, K13	907	[74]
**KP32**	NC_013647.1	*Autographiviridae*	41,119	52.4	**KP32gp37**	YP_003347555.1	K3	869	[71]
**K5-4**	KY389316.1	*Autographiviridae*	40,163	53.1	**ORF38**	APZ82848.1	K5	684	[16]
**P1011**	OR492660	*Drexlerviridae*	49,460	50.4	**dep1011**	WNO47121.1	K5	857	[75]
**vB_KpnM-20**	MZ826764.1	*Ackermannviridae*	158,784	46.5	**K7dep**	UCR74082.1	K7	1180	[47]
**K5-4**	KY389316.1	*Autographiviridae*	40,163	53.1	**ORF37**	APZ82847.1	K8	749	[16]
**K64-1**	NC_027399.1	*Alcyoneusvirus*	346,602	31.7	**S1-1**	BAW85694.1	K11	702	[17]
**SH-KP156570**	^**^	*Autographiviridae*	39,997	50.7	**Dpo41**	N.A	K19	763	[76]
**vB_KpnM-20**	MZ826764.1	*Ackermannviridae*	158,784	46.5	**K20dep**	UCR74085.1	K20	723	[47]
**Klyazma**	OP125547.1	*Zobellviridae*	50,298	46.0	**Kl-dep**	UVD31996.1	K20	790	[77]
**K5**	NC_0228800.1	*Autographiviridae*	41,698	52.5	**K5-RBP2**	YP_009198669.1	K21	575	[78]
**KP32**	NC_013647.1	*Autographiviridae*	41,119	52.4	**KP32gp38**	YP_003347556.1	K21	576	[71]
**K64-1**	NC_027399.1	*Alcyoneusvirus*	346,602	31.7	**S1-3**	BAW85693.1	K21	651	[17]
**Dlv622**	MT939252.1	*Autographiviridae*	44,687	54.0	**Dlv622_00059**	QOI68577.1	K23	555	[11]
**KpS8**	NC_048873.1	*Vequintavirinae*	143,800	44.6	**Kps8_053**	YP_009859099.1	K23	607	[11]
**K64-1**	NC_027399.1	*Alcyoneusvirus*	346,602	31.7	**S2-2**	BAW85696.1	K25	584	[17]
**vB_KpnM-20**	MZ826764.1	*Ackermannviridae*	158,784	46.5	**K27dep**	UCR74083.1	K27	1294	[47]
**K64-1**	NC_027399.1	*Alcyoneusvirus*	346,602	31.7	**S2-6**	BAW85699.1	K30, K69	767	[17]
**K5-2**	KY389315.1	*Autographiviridae*	41,116	47.5	**ORF37**	APZ82804.1	K30, K69	792	[16]
**K64-1**	NC_027399.1	*Alcyoneusvirus*	346,602	31.7	**S2-3**	BAW85697.1	K35	779	[17]
**IME205**	KU183006.1	*Autographiviridae*	41,310	52.2	**Dpo43**	ALT58498	K47^*^	641	[48]
**IME205**	KU183006.1	*Autographiviridae*	41,310	52.2	**Dpo42**	ALT58497	K47^*^	793	[48]
**P560**	MT966873.1	*Autographiviridae*	40,562	53.1	**P560dep**	QOV05502.1	K47	641	[49]
**SH-KP152226**	MK903728.1	*Autographiviridae*	41,420	52.7	**Dep42**	QDF14644.1	K47	793	[24]
**GBH019**	OU509535.1	*N.I*	347,546	32.0	**GBH019_279**	N.A	K51	809	[54]
**RaK2**	NC_019526.1	*Alcyoneusvirus*	345,809	31.7	**RaK2gp531**	YP_007007686.1	K54	895	[42]
**KN3-1**	LC413194.1	*Autographiviridae*	41,059	53.5	**K56dep**	BBF66868.1	K56	678	[79]
**ZX1**	MW722080.1	*Lastavirus*	60,982	57.0	**Dep_ZX1**	QTH79846.1	K57	614	[80]
**KpV79**	MF663761.1	*Jedunavirus*	47,760	49.0	**Dep_kpv79**	ATI16495.1	K57	721	[13]
**KpV767**	KX712070.1	*Autographiviridae*	40,395	52.3	**Dep_kpv767**	AOZ65519.1	K57	843	[13]
**SH-KP2492**	OR700190.1	*Drexlerviridae*	48,383	50.3	**K62-Dpo30**	WOK01638.1	K62	692	[81]
**KP34**	NC_013649.2	*Autographiviridae*	43,809	54.0	**KP34gp57**	YP_003347651.1	K63	630	[32]
**KP36**	NC_029099.1	*Drexlerviridae*	49,797	50.7	**depoKP36**	YP_009226011.1	K63	883	[82]
**K64-1**	NC_027399.1	*Alcyoneusvirus*	346,602	31.7	**S2-5**	BAQ02780.1	K64	996	[17]
**P510**	MT966872.1	*Autographiviridae*	39,383	53.1	**P510dep**	QOV05454.1	K64	1017	[53]
**SH-Kp 152410**	MG835568.1	*Autographiviridae*	40,945	52.4	**K64-ORF41**	AUV61507.1	K64	1017	[58]
**IME321**	MH587638.1	*Autographiviridae*	39,906	52.8	**Dp42**	AXE28435	KN1	820	[83]
**KN1-1**	LC413193.1	*Autographiviridae*	40,236	52.8	**KN1dep**	BBF66844.1	KN1	820	[79]
**0507-KN2-1**	AB797215.1	*Ackermannviridae*	159,991	46.7	**ORF96**	BAN78446.1	KN2	1245	[84]
**KN3-1**	LC413194.1	*Autographiviridae*	41,059	53.5	**KN3dep**	BBF66867.1	KN3	792	[79]
**K64-1**	NC_027399.1	*Alcyoneusvirus*	346,602	31.7	**S1-2**	BAW85692.1	KN4	736	[17]
**KN4-1**	LC413195.1	*Autographiviridae*	41,219	53.0	**KN4dep**	BBF66888.1	KN4	850	[79]
**K64-1**	NC_027399.1	*Alcyoneusvirus*	346,602	31.7	**S2-1**	BAW85695.1	KN5	1193	[17]
**KPPK108.1**	OK583892.1	*Autographiviridae*	43,755	53.6	**Dep108.1**	UFK09522.1	KN8	590	[68]
**KPPK108.2**	OK583892.1	*Autographiviridae*	43,834	54.0	**Dep108.2**	UZN24616.1	KN8	595	[68]

All 58 experimentally validated *K. pneumoniae* phage depolymerases are shown, ordered by CPS target. Summary includes the phage origin of the depolymerase, phage family, phage genome size and GC content of phage, CPS target, and depolymerase protein length.

N.I = Not Identified. N.A = Not Available.

* Dpo42 and Dpo43 target two different subsets of K47.

** Phage SH-KP156570 can be found on https://ngdc.cncb.ac.cn/gwh/ via accession number GWHBGZN00000000.

**Table 3:** Identified CPS cleavage sites of *K. pneumoniae* phage depolymerases

Depolymerase	Depolymerase accession number	CPS serotype	Cleaved bond	Activity type	Reference
**NTUH-2044-K1-1 ORF34**	YP_009098385.1	K1	β-D-Glc-(1→4)-β-D-GlcA	Lyase - Beta Elimination	[18]
**DpK2**	QUU29414.1	K2	α-D-Glc-(1→3)-β-D-Glc	Hydrolase - N.I	[8]
**K2-2**	QJI52632	K2	β-D-Glc-(1→4)-β-D-Man	Hydrolase - Retaining	[39]
**K2-ORF16**	ATS92567.1	K2	β-D-Glc-(1→4)-β-D-Man	Hydrolase - N.I	[71]
**K5-RBP2**	YP_009198669.1	K21	β-D-Gal-(1→3)-α-D-GlcA	Hydrolase - N.I	[78]
**RaK2gp531**	YP_007007686	K54	β-D-Glc-(1→4)-α-D-GlcA	Hydrolase - N.I	[42]
**Dep_kpv767**	AOZ65519.1	K57	β-D-Gal-(1→3)-α-D-GalA	Hydrolase - N.I	[13]
**Dep_kpv79**	ATI16495.1	K57	β-D-Gal-(1→3)-α-D-GalA	Hydrolase - N.I	[13]
**KP34gp57**	YP_003347651.1	K63	α-D-Gal-(1→3)-α-D-GalA	Hydrolase - N.I	[32]
**Depo108.1**	UFK09522	KN8	β-Glc-(1→4)-α-Fuc	Hydrolase - N.I	[68]
**Depo108.2**	UZN24616	KN8	β-Glc-(1→4)-α-Fuc	Hydrolase - N.I	[68]

All eleven experimentally validated CPS cleavage sites are shown, ordered by CPS target. Summary includes the sugar residues adjacent to the cleaved bond, and the activity type of the enzyme. α or β denotes the sugar anomer, while D or L denotes stereoisomerism. Accession numbers provided correct for NCBI GenBank at time of writing.

N.I = Not Identified.

### Depolymerases can demonstrate extended or reduced substrate specificity

**Figure 4:**
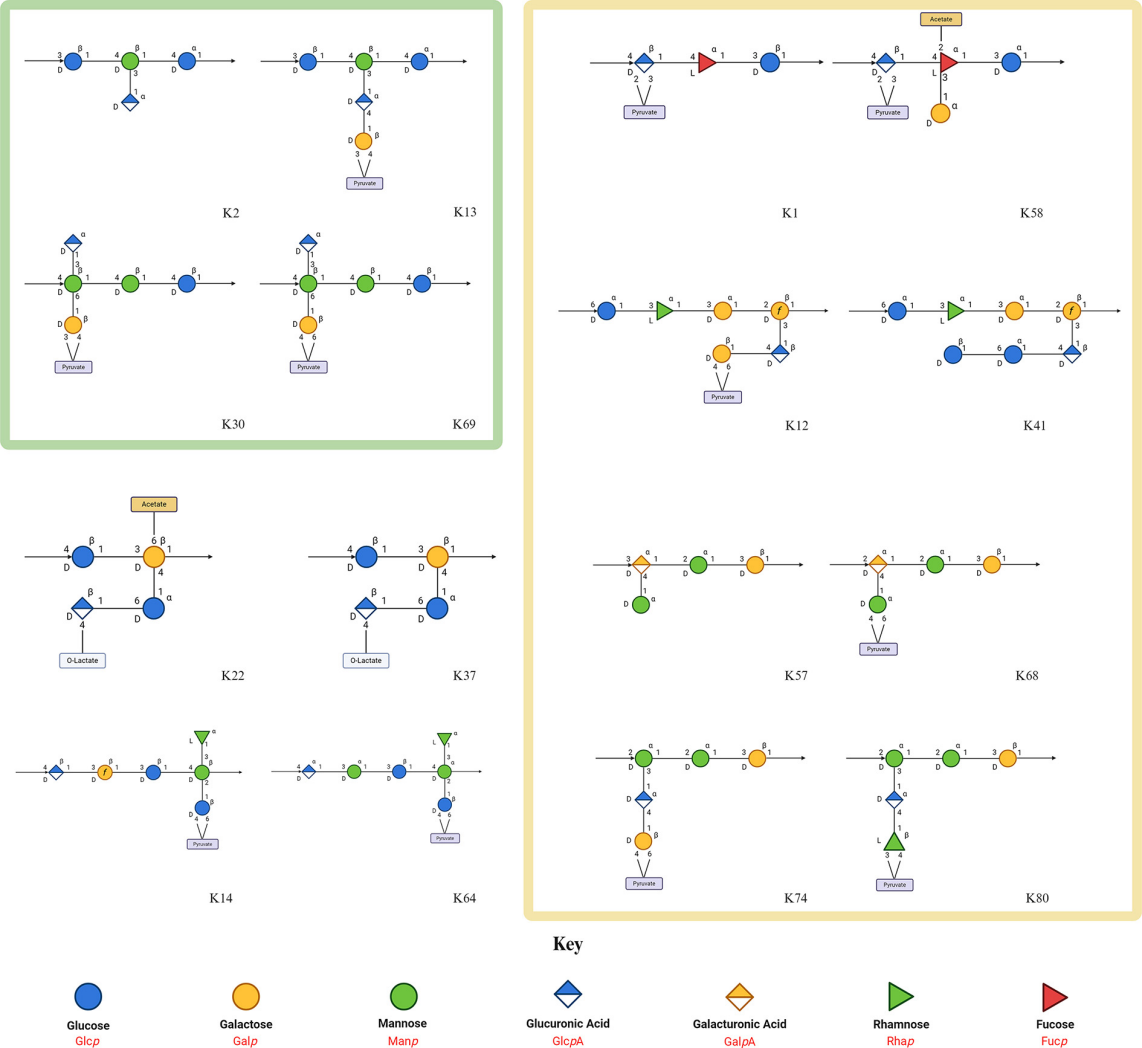
Structures of similar *Klebsiella* capsule polysaccharides Schematic representations of capsule polysaccharide repeat unit structures were sourced from K-PAM (https://www.iith.ac.in/K-PAM/k_antigen.html) and are arranged side-by-side in similar pairs. Similar structures with depolymerases known to target both are highlighted by a green outline. Similar structures that have been proposed previously [50] are highlighted in yellow. A glycan key is included.

The recognition, binding, and degradation mechanisms employed by polysaccharide depolymerases gives rise to a large variation in specificities, in response to the diversity of encountered substrates [26]. This is often associated with a narrow host range observed in *Klebsiella* phage depolymerases, such as Dpo16, S2-4 and Kpv71_52 only targeting K1 (Table 2). It must be noted, however, that estimates of specificity are entirely dependent on rigorous testing, which is at the discretion of individual researchers and yet to be standardised. Despite this, depolymerases demonstrating extended substrate specificity have been observed (Table 2). Both K30-degrading depolymerases, S2-6 from phage K64-1 and ORF37 from phage K5-2, can also degrade the K69 CPS, as demonstrated by two independent studies [16,17]. This can be explained by highly similar chemical compositions between the two serotypes, differing only by a pyruvyl linkage to a galactose moiety (Figure 4). As such, it is theorised both enzymes recognise the common CPS backbone, rendering the differing pyruvyl positions irrelevant for determining specificity. Similarly, the K2 depolymerases that have been tested on K13 (Kpv74_56, K2-2 and B1dep) were capable of degrading both K2 and K13 CPS. Akin to K30 and K69 capsules, the K2 and K13 capsules share highly similar structures (Figure 4), and it’s likely that the additional pyruvylated-galactose moiety in K13, plays a minor role in CPS recognition by K2/13 depolymerases. As the remaining K2 depolymerases were not tested against K13, it cannot be concluded that all K2 depolymerases have K13 activity. Extended substrate specificity was also observed for depolymerase KP34gp57, which targets the K63 serotype in *K. pneumoniae*, and the identical K42 serotype in *E. coli*, highlighting possible depolymerase cross-reactivity between bacterial species with similar capsule structures [32].

**Figure 5:**
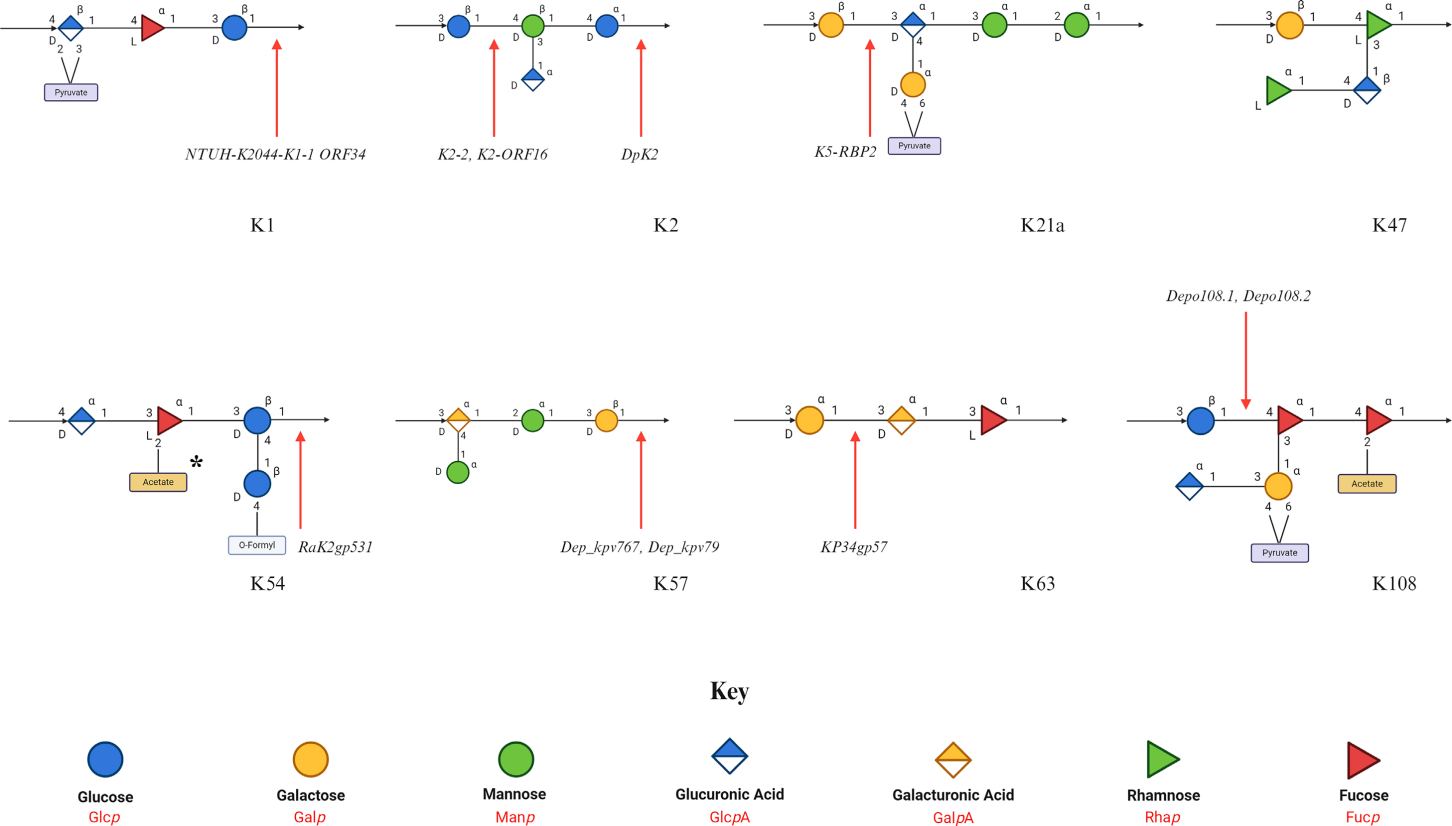
Structures and cleavage sites of *Klebsiella* capsule polysaccharides Schematic representations of capsule polysaccharide repeat unit structures were sourced from K-PAM (https://www.iith.ac.in/K-PAM/k_antigen.html) with the exception of K108 sourced from [68]. Identified cleavage sites (see Table 3) of CPS structures shown by a red arrow. A glycan key is included. * 2-Acetylation on the Fucose of K54 appears every other repeat unit.

Reduced substrate specificity can be observed in the case of K47 depolymerases from phage IME205 that only target one of two capsular subsets [48]. Phage IME205 could degrade all K47 CPS strains it was tested on, and two depolymerases were isolated from from this phage: Dpo42, showing activity on 16/56 strains, and Dpo43 the remaining 40/56, forming a different K47 subset. This phenomenon has also been observed with another experimentally validated K47 depolymerase, P560dep, which could only degrade the CPS of one subset of assayed K47 *K. pneumoniae* strains (19/24) [24,49]. The K47 capsular subsets are likely differentiated by subtle structural modifications, which cannot be identified by gene sequencing alone, and impact K47 depolymerase activity [48]. While serological studies have deduced the K47 capsule structure (Figure 5), the hypothesised capsular modifications have not yet been identified.

### Capsule similarity (whole structure or cleaved glycosidic bonds) is not always sufficient for extended-substrate depolymerases

Due to limited data, the extended-substrate functionality across K13- and K30-targeting depolymerases cannot yet be concluded as ubiquitous. Such observations, however, should prompt further investigation, as CPS structural similarities may enable other depolymerases to target multiple capsule types. Analysis of the known CPS structures suggests other pairs of similar K-types, against which only one depolymerase may be sufficient. These include: K1 and K58, K12 and K41, K57 and K68, K74 and K80 [50], K37 and K22 [51], and K64 and K14 (Figure 4). Identified K1 depolymerases, however, show no activity against tested K58 strains [17,52]. Similarly, K64 depolymerases show no activity against K14 [17,53]. Many cases, however, lack sufficient host-range testing. For example, it is unclear whether all the identified K57 depolymerases have been tested on K68 strains, and some K1 and K64 depolymerases have not been tested on K58 and K14 strains, respectively [54–58]. Depolymerases against K12, K41, K74, K80, K37 and K22 are yet to be described.

Identification of the cleaved bond does not appear to be sufficient for prediction of capsule recognition by the depolymerase. That is, enzymes targeting similar glycosidic bonds such as K21-targeting K5-RBP2 and K57-targeting Dep_kpv79 (cleaving β-D-Gal-(1→3)-α-D-GlcA, and β-D-Gal-(1→3)-α-D-GalA, respectively) maintain their capsular specificity, despite the neighbouring sugars differing only by one hydroxyl position (GalA has carbon-4 pointing up, where GlcA has carbon-4 pointing down). This suggests that a larger region of the CPS is necessary for enzymatic recognition. It follows, therefore, that some regions of the CPS may be less important for recognition, as perhaps illustrated by K30/69 depolymerases (seemingly ignoring the pyruvyl positioning) and K2/13 depolymerases (seemingly ignoring the additional pyruvylated-galactose in K13) [14,16,17,39,59]. Similarly, structural characterisation looking at the electrostatic potential of solvent-accessible grooves in depolymerases specific to dissimilar substrates shows that they have distinct structural characteristics, even when the catalytic residues are conserved, such as in the case of the K1 and K64 lyases (NTUH-K2044-K1-1 ORF34 and K64-ORF41, respectively) [33]. Together, this may imply that ‘recognition motifs’, specific moieties within the CPS, are crucial for recognition by a specific depolymerase, in addition to the presence of particular glycosidic bonds to facilitate cleavage. Future research aiming to isolate and characterise CPS depolymerases should ensure broader host range testing to determine the depolymerase enzymatic spectrum. Using detailed 3D alignments of different capsules may allow for more accurate assessment of depolymerase recognition and activity.

## Structural and phylogenetic conservation of *Klebsiella* phage-encoded depolymerases

### Depolymerase N-terminal domain conservation

**Figure 6:**
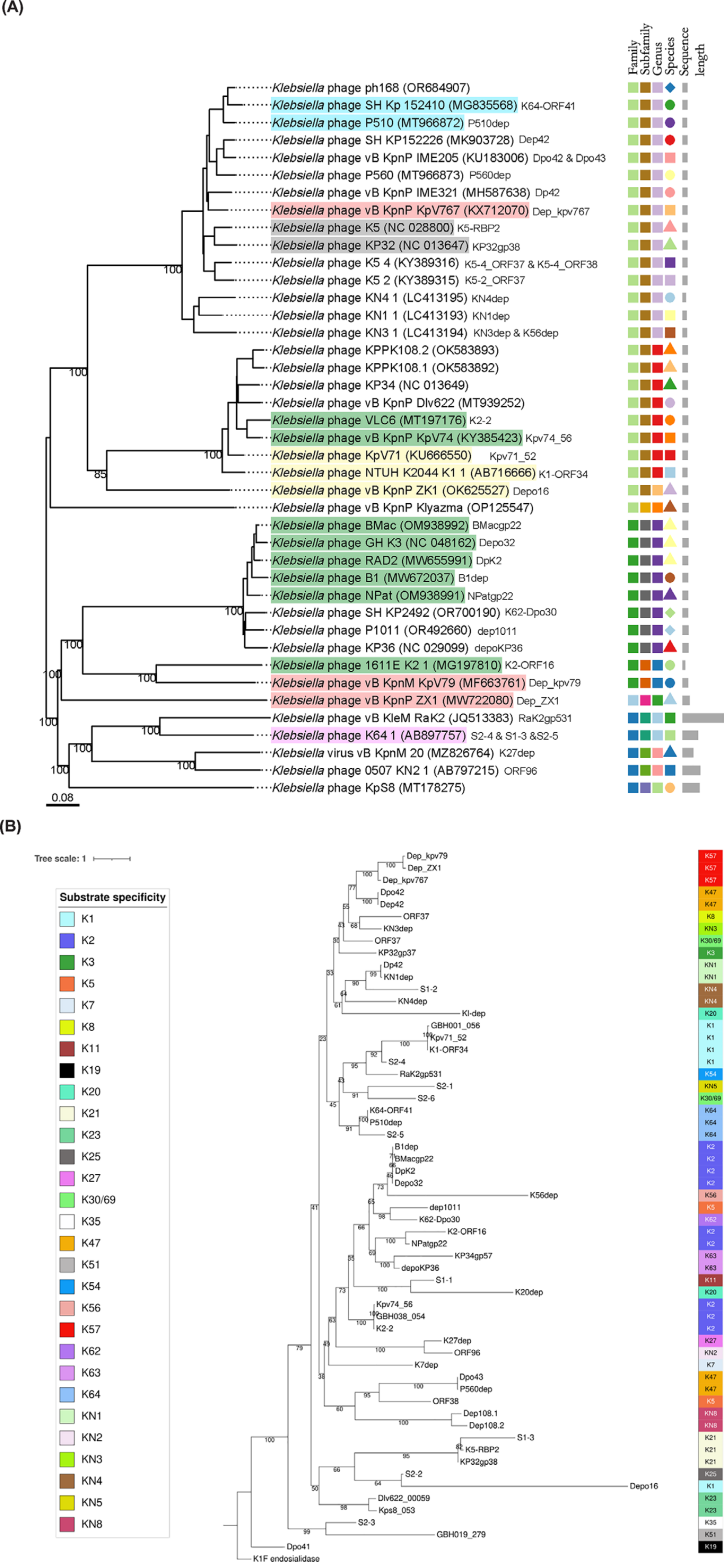
Phylogenetic analysis of experimentally validated *Klebsiella* CPS depolymerases and the phages which encode them (**A**) Phylogenetic tree of phages encoding experimentally validated depolymerases. The tree was generated using all phage CDS in VICTOR, a method for the genome-based phylogeny and classification of prokaryotic viruses [69,70]. Phages encoding depolymerases discussed in the text are highlighted: K21 phages (grey), K64 phages (blue), K57 phages (red), K2 phages (green), K1 phages (yellow), and phage K64-1 (purple) that encodes K1, K21 and K64 depolymerases. Depolymerase names are listed alongside their encoding phages for easier interpretation (**B**) Phylogenetic tree of 58 experimentally validated *Klebsiella* phage-encoded CPS depolymerases, and the *E. coli*-targeting phage K1F depolymerase as an outgroup. The entire depolymerase sequence was used and aligned using T-Coffee, a structure-based consensus alignment. Capsule specificity determines the node colours (legend shown).

Despite the broadly conserved architecture observed in all *Klebsiella* phage depolymerases identified to date, the effects of intensive vertical and horizontal evolution is evident in the sequence diversity and mosaicism of the genes encoding depolymerase-containing RBPs [60]. The role of modularity and horizontal gene transfer has been highlighted as key to rapid adaptation in the face of *K. pneumoniae* capsular diversity and phenotypic serotype switching, suggesting that conservation in regions encoding for depolymerase tethering should be reflective of phylogenetic relationships [12]. The mechanisms of organisation and RBP tethering to *Klebsiella* phages was previously explored [12] highlighting how the modularity of RBPs can be exploited by extensive horizontal gene transfer, with NTD conservation allowing for the dynamic re-use of protein domains [26]. When comparing protein sequences of the experimentally validated depolymerases, conservation of the NTD between depolymerases targeting different capsule types is apparent, all from phages in the same genus (e.g. KP32gp37, KN3dep, Dep42, Dpo43, K5-4 ORF37, K5-2 ORF37, K64-ORF41, P510dep, KN1dep, Dp42, KN4dep, Dep_kpv767) (Figure 6A). This is consistent with the proposed model of RBP system organisation within the KP32virus [12]. Different tethering mechanisms may be employed by phages within the same genus, as illustrated by the variation in NTD conservation (e.g. between Dep42 and P560dep), consistent with observed anchor/branch attachment variations within groups, and highlighting the role of the NTD depolymerase-phage tethering and modularity (more detailed analysis was outside of the scope of this review) [26].

### Depolymerases display structural diversity when targeting different or similar capsule structures

Previously, studies have shown that phage encoded CPS-, LPS-, and EPS-degrading depolymerases demonstrate clustering partly based on substrate specificity [25]. Understanding similarity between *Klebsiella* depolymerases that target the same CPS substrate has not, to the best of our knowledge, been rigorously studied, yet it could have key implications for predicting host range *in silico*. We included all 58 experimentally validated depolymerases in our analysis (Figure 6B), supplementing the solved structural comparisons (Figure 2A).

Unsurprisingly, depolymerases specific to different substrates display different structural features. Namely, when comparing structurally characterised depolymerases targeting different CPS serotypes, low protein sequence identity (<25%) is observed (Figure 2B). Similarly, the phylogenetic analysis of the entire protein sequence (including the NTD) of the 58 depolymerases reveals instances of clustering based on substrate-specificity (Figure 6B). To account for depolymerase similarity largely resulting from parent phage phylogeny (e.g., high levels of conservation only in the NTD, or Depo32 and DpK2 sharing > 98% sequence identity), here we only discuss depolymerases that: (i) target the same capsule type, (ii) have more than two representatives, and (iii) derive from phages belonging to dissimilar genera. K21-, K57-, and K64-specific depolymerases demonstrate sub-clustering based on sequence identity (Figure 6B), and show that depolymerases from more distantly related phages share depolymerase sequence identity predominantly in the catalytic and C-terminal domains (e.g., for K21 depolymerases: closely related phages K5 and KP32, both in the genus *Przondovirus*, have depolymerases K5-RBP2 and KP32gp38 that share 87% identity in 100% QC, compared to 38% identity in 95% QC for depolymerase S1-3, encoded by the distantly related phage K64-1 in the genus *Alcyoneusvirus*) (Figure 6A). In other instances, evidence of sub-groups based on sequence identity and structural features emerging within substrate-defined classes of depolymerases was apparent, such as in the case of K1- and K2-specific depolymerases (Figure 6B). Depolymerases targeting K2 capsules are the most numerous of those experimentally validated, and they can be assigned to three sub-groups based on whole-sequence comparisons: group 1, including BMacgp22, Depo32, DpK2, and B1dep and group 2, consisting of K2-ORF16 and NPatgp22, and group 3, with GBH038_054, Kpv74_56, and K2-2 (Figure 6B). In both the K1- and K2- cases, phage phylogeny appears to play a role in depolymerase sub-clustering (Figure 6A).

The solved structures of K2-depolymerases Depo32 and K2-2 show only share 16% amino acid identity (0.55 TM-Score) (Figure 2B) and differ in their catalytic mechanism (hydrolysis by inverting and retaining, respectively) (Figure 3A, B). The glycosidic bonds cleaved by representatives of K2 depolymerase sub-groups also vary; DpK2 hydrolyses the α-D-Glc-(1→3)-β-D-Glc glycosidic bond in the K2 CPS, yet K2-targeting depolymerases K2-ORF16 and K2-2 both hydrolyse a different glycosidic bond, β-D-Glc-(1→4)-β-D-Man (Figure 5). Whether these are instances of possible evolutionary convergence remains to be seen, however in-depth comparison of conserved residues across sub-groups could be important for elucidating the mechanisms involved in CPS recognition. Interestingly, protein family subgroups that have different substrate specificities have also been observed, such as in the case of polysaccharide lyases K64-ORF41 and NTUH-K2044-K1-1 ORF34 (K64 and K1, respectively), and Gp54, which depolymerizes *A. baumannii* CPS [33,61]. The overall structural similarity, with specialised CPS binding domains, suggests possible evolution of divergence in response to diverse substrates. This also illustrates the importance of detailed characterisation, as structural comparison enabled identification of conserved catalytic residues across the three enzymes, with important implications for the possibility of engineering multi-serotype specificity. It is likely, therefore, that different structural depolymerase ‘families’ exist against each serotype, further diversifying *Klebsiella*-specific depolymerases and their respective mechanisms [41]. This would be in line with what has been observed more broadly within polysaccharide lyases and glycosyl hydrolases, where families and sub-families have been proposed based on similarities in amino acid sequence identity [41,62–64]. Enzymes with different substrate specificities found in the same family could indicate an evolutionary divergence to acquire new specificities. Also, enzymes that act on the same substrate sometimes in different families, point to possible evolutionary convergence [41]. This highlights the need for further structural characterisation of validated depolymerases, to expand our understanding of underlying CPS depolymerase classification in *Klebsiella* phages. The fact that the evidence for sub-groups within same-substrate depolymerases correlates with the number of depolymerases identified for that specific substrate, indicates that we are likely only scratching the surface of *Klebsiella* CPS depolymerase complexity. This has important implications for the development of strategies predicting depolymerase specificity based on protein sequence identity.

## Summary

- The rise of MDR *K. pneumoniae* has sparked renewed interest in *Klebsiella* phages, and detailed characterisation of capsular depolymerases is crucial for both understanding (and predicting) phage activity, as well as for developing stand-alone depolymerase therapeutics.
- *Klebsiella* depolymerases are structurally and mechanistically diverse. They can be either lyases or hydrolases (both retaining and inverting) and cleavage can occur at the inter- and intra- subunit locations, which has important implications for engineering recombinant proteins with a reduced size and broader spectrum.
- Depolymerases can demonstrate extended substrate specificity, largely due to similarity of capsule structures. This highlights the need to further investigate common recognition motifs, to determine the true range of depolymerase activity.
- Depolymerase structural diversity doesn’t accurately reflect substrate specificity, suggesting different depolymerase ‘sub-families’, based on sequence and structural alignment, which we suggest shouldn’t be species-restricted. This indicates that enzymatic specificity prediction *in silico* requires further research and highlights the role of divergence and convergence in shaping depolymerase diversity.
- Furthur research should aim to: (1) explore methods of sufficient host-range testing, to determine if isolated depolymerases display extended-substrate functionality, (2) structurally characterise depolymerases and determine their mechanisms of recognition and catalysis, (3) discover additional depolymerases and compare them with the existing depolymerase library to offer valuable insights into the evolution of depolymerases and the diversity of their catalytic mechanisms.

## Open Access

Open access for this article was enabled by the participation of University College London in an all-inclusive *Read & Publish* agreement with Portland Press and the Biochemical Society under a transformative agreement with JISC.

## Abbreviations

AA: amino acid
CPS: capsule polysaccharide
CTD: C-terminal domain
EPS: extracellular polysaccharide
Gal: galactose
GalA: galacturonic acid
Glc: glucose
GlcA: glucuronic acid
LPS: lipopolysaccharide
Man: mannose
MDR: multidrug resistant
NTD: N-terminal domain
QC: query cover
RBP: receptor binding protein
TM: template modelling
